# Supplementary material for: Bound States in the Continuum Active Metasurfaces for Tunable Amplified Photoluminescence and Single-Photon Emission
Source: Nanomaterials (Basel). 2026 Aug 12;16(16):994. doi: 10.3390/nano16160994 (PMC13515580; doi:10.3390/nano16160994)
Supplement: Supplementary file 1 [file nanomaterials-16-00994-s001.zip › nanomaterials-4448389-supplementary.pdf]

Supporting Information:

Bound States in the Continuum Active Metasurfaces for Tunable Amplified Photoluminescence and Single-Photon Emission

Omar A. M. Abdelraouf [1, 2]

Affiliations:

[1] Engineering Physics and Mathematics Department, Faculty of Engineering, Ain Shams University, Cairo 11535, Egypt

[2] Institute of Materials Research and Engineering, Agency for Science, Technology, and Research (A*STAR), 2 Fusionopolis Way, #08-03, Innovis, Singapore 138634, Singapore

Corresponding author: Omar A.M. Abdelraouf (email: omar.abdelraouf@eng.asu.edu.eg)

Table of Contents:

S1. Measured refractive indices used in tunable BIC metasurfaces

S2. Purcell Factor Calculation and Optical Mode Volume Analysis

S3. Fabrication flow of tunable BICs metasurface

S4. Photoluminescence of $CuInSe_2$ (CIS) QDs before and after annealing

S5. Photoluminescence measurement setup

S6. Benchmark with metasurfaces for tunable amplified PL and SPE

S1. Measured refractive indices used in tunable BIC metasurfaces

Figure S1 shows the refractive indices of used materials in fabricating the tunable BIC metasurface, measured by variable angle ellipsometry.

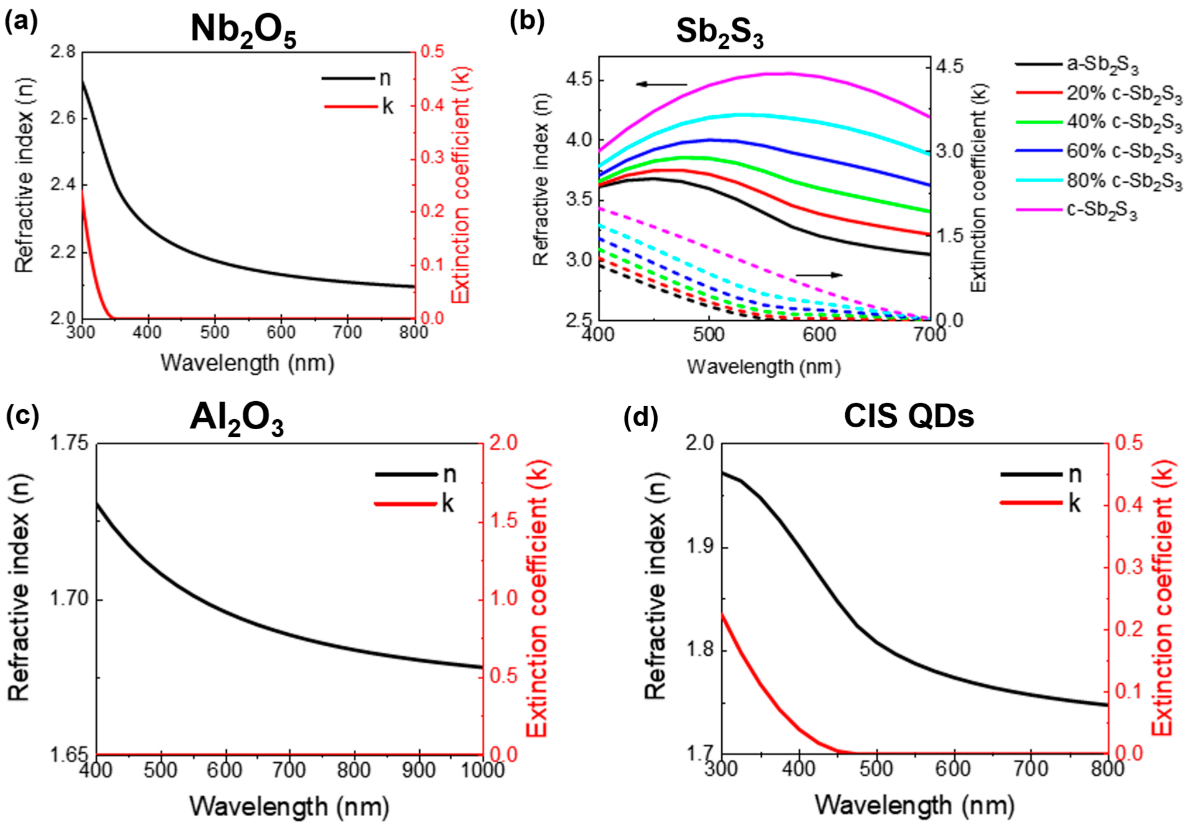

Figure S1. Measured refractive index of materials in the tunable BIC metasurface. (a) amorphous niobium pentoxide (Nb_2O_5). (b) antimony trisulfide in the amorphous state (a-Sb_2S_3) and various degrees of the crystalline states (c-Sb_2S_3). (c) aluminum dioxide (Al_2O_3). (d) Copper Indium Selenide $CuInSe_2$ (CIS) QDs.

S2. Purcell Factor Calculation and Optical Mode Volume Analysis

The Purcell factor was calculated to quantify the spontaneous emission enhancement induced by the resonant dielectric metasurface quasi-BIC. The enhancement originates from the simultaneous realization of a high optical quality factor and strong electromagnetic field confinement inside the CIS QDs surrounded by Nb_2O_5 nanoresonators. The Purcell factor was evaluated according to the classical cavity quantum electrodynamics (cQED) expression.

$$F_P = \frac{3}{4\pi^2}\left(\frac{\lambda}{n}\right)^3 \frac{Q}{V_{\text{eff}}} \tag{S1}$$

where F_p is the Purcell enhancement factor, λ is the free-space resonance wavelength, n is the refractive index at the resonance wavelength, Q is the simulated quality factor of the resonant mode, and V_{eff} *is* the effective optical mode volume. The effective mode volume was determined from the spatial distribution of the electric field using:

$$V_{\text{eff}} = \frac{\int \varepsilon(\mathbf{r})|E(\mathbf{r})|^2 dV}{\max[\varepsilon(\mathbf{r})|E(\mathbf{r})|^2]} \tag{S2}$$

Where $\varepsilon(r)$ is the local dielectric permittivity and E(r) is the electric field amplitude. The volume integration was performed only inside the Nb_2O_5 nanoresonators by applying a three-dimensional material mask generated from the refractive index distribution. Air and substrate regions were excluded from the integration to ensure that the calculated mode volume represents the optical confinement within the active resonator rather than the entire simulation domain. The electric field distributions were obtained using three-dimensional FDTD simulations under broadband normally incident plane-wave excitation. The absorbed electromagnetic energy was calculated as:

$$P_{\text{abs}} = \frac{1}{2}\int |E(\mathbf{r})|^2 \text{Im}[\varepsilon(\mathbf{r})] dV \tag{S3}$$

which was integrated over the entire computational domain. The Purcell enhancement was subsequently calculated using the simulated quality factor extracted from the quasi-BIC resonance together with the effective mode volume obtained at each wavelength. The calculations reveal strong light confinement inside the dielectric resonator as shown in Figure S2, the amorphous amorphous metasurface exhibits a maximum Purcell factor of approximately 156.

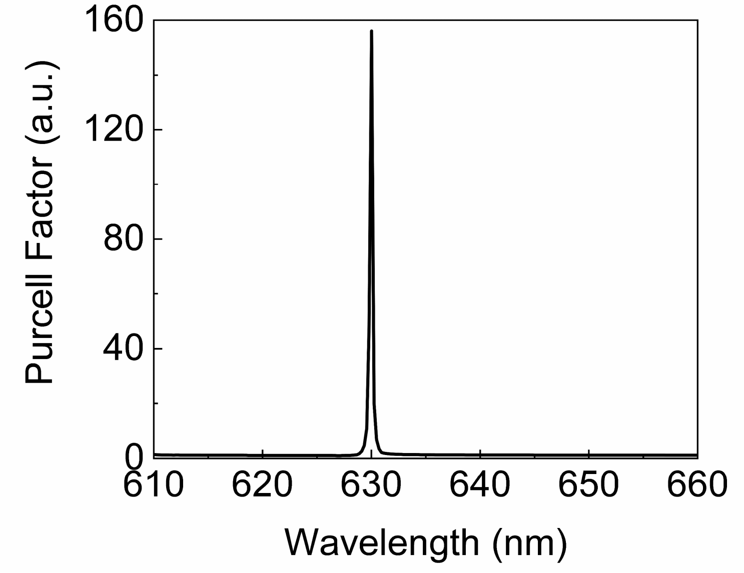

Figure S2. Simulated Purcell factor spectra of the quasi-BIC metasurface for the amorphous states.

S3. Fabrication flow of tunable BICs metasurface

Figure S3 shows the fabrication flow of tunable amplified classic and quantum light emission in the visible using a Nb_2O_5 BIC metasurface.

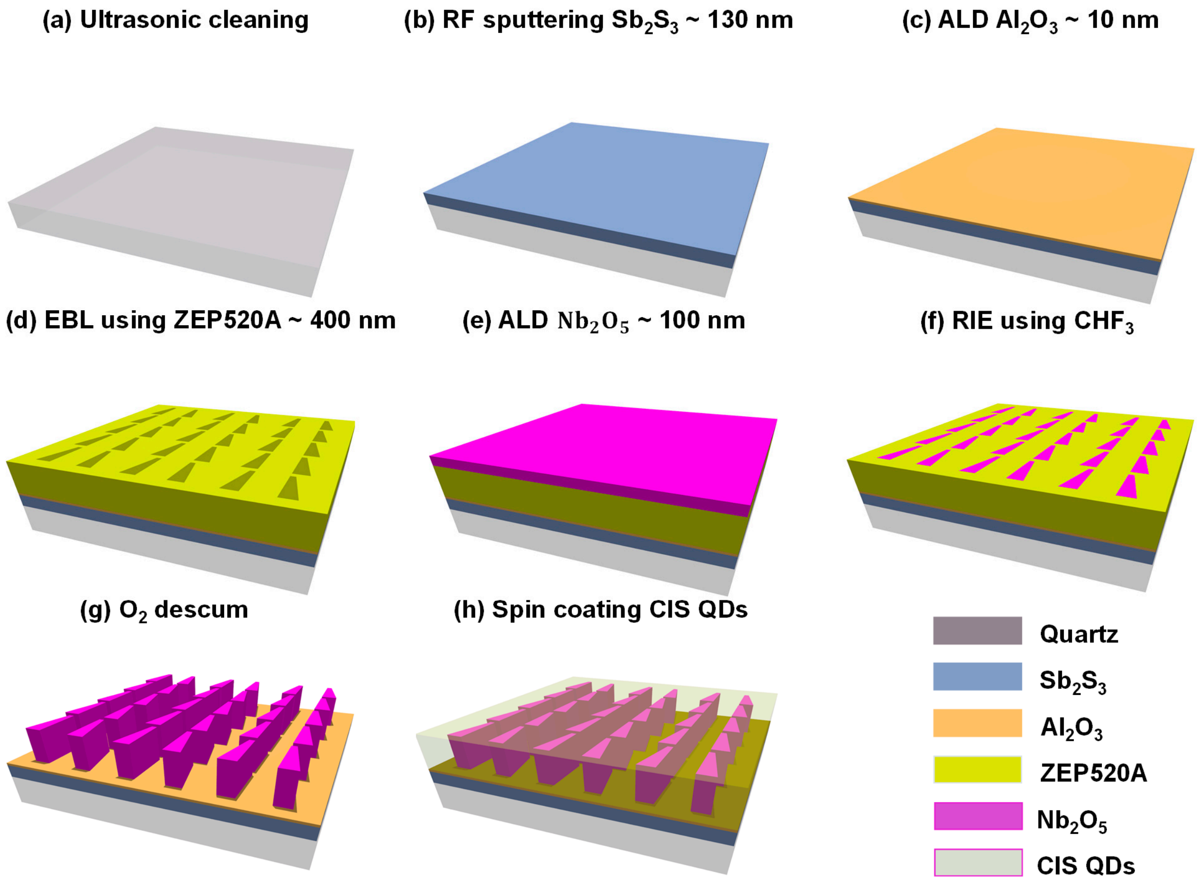

Figure S3. Fabrication flow of the active hybrid BIC metasurface.

S4. Photoluminescence of CuInSe2 (CIS) QDs before and after annealing

The photoluminescence spectrum of CIS QDs after spin coating at 4k rpm on silicon substrate is shown in Fig. S4 and using same cw-laser power of wavelength 532 nm before and after annealing at 300 C° for 3 minutes.

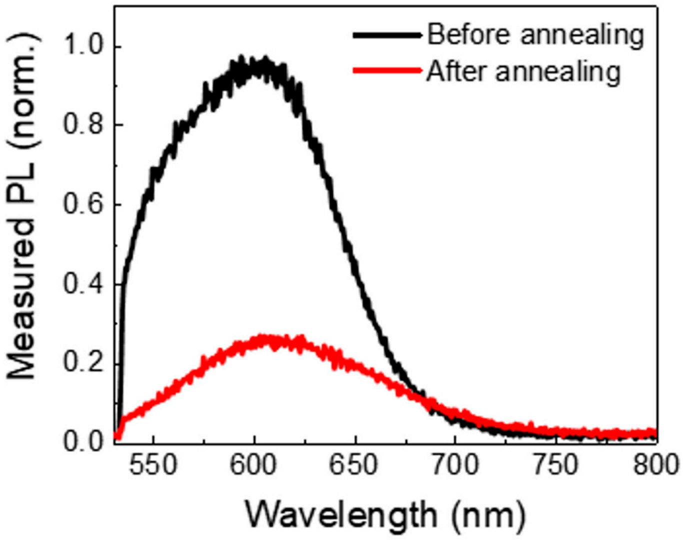

Figure S4. Measured photoluminescence of CIS QDs thin film before and after annealing.

S5. Photoluminescence measurement setup

The optical setup used in all PL measurements of resonant metasurface cavities is shown in Fig. S5. The detector, spectrometer, and a fiber-coupled continuous-wave (CW) laser at a wavelength of 532 nm were used from a WITec system with an average power of up to 50 mW.

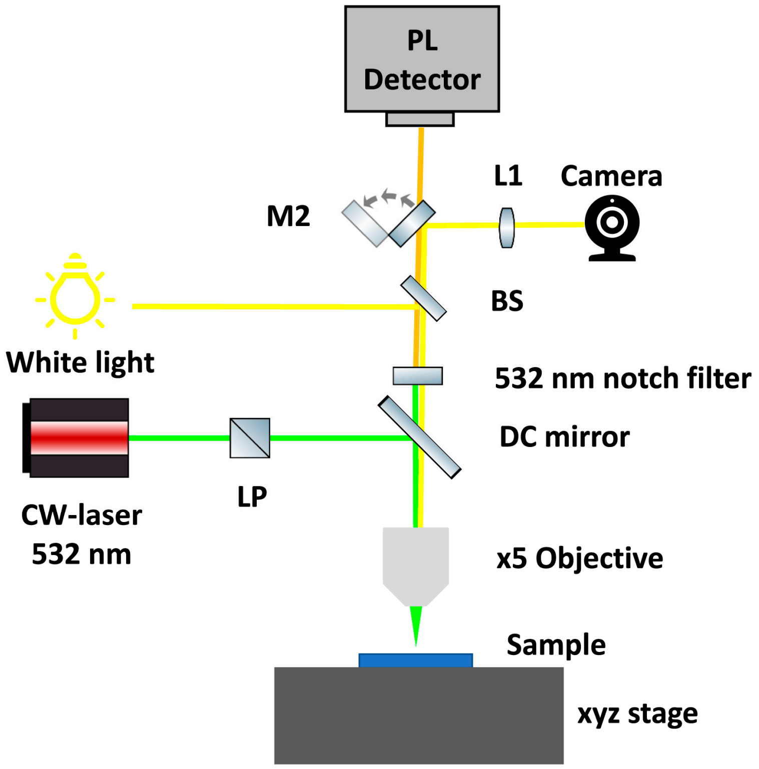

Figure S5. Optical setup used for photoluminescence measurements.

S6. Benchmark with metasurfaces for tunable amplified PL and SPE

A comprehensive performance comparison between our active BIC hybrid metasurface and existing state-of-the-art metasurfaces from the literature was conducted, evaluating key metrics including *Q*-factor, photoluminescence amplification factor, tuning range, and single-photon emission (SPE) enhancement, as detailed in Table S1. Our active BIC hybrid metasurface demonstrates exceptional performance characteristics that surpass current benchmarks in the field. The enhanced BIC resonance configuration achieved an experimental *Q*-factor reaching 206, establishing strong electromagnetic field confinement. Most notably, we achieved the highest reported photoluminescence amplification factor exceeding 33-fold enhancement compared to reference samples. The platform exhibits remarkable spectral tunability with over 17 nm tuning range through dimensional parameter variation, complemented by 28 nm wavelength shift capability via Sb_2S_3 phase transitions. Furthermore, we successfully demonstrated on-demand strong coupling for amplified single-photon emission with enhancement factors exceeding two orders of magnitude, positioning our hybrid metasurface as a leading platform for quantum nanophotonic applications.

Table S1. Comparison of metasurfaces in literature for tunable amplified PL and SPE.

	**Our work**	**Ref [1]**	**Ref [2]**	**Ref [3]**	**Ref [4]**	**Ref [5]**	**Ref [6]**	**Ref [7]**	**Ref [8]**
**Metasurface resonance**	BIC	BIC	BIC	Dual BIC	Plasmo nic BIC	Hyperbo lic	Grating	No	Gap plasmon
**Metasurface Q-factor**	206	106	2089	208 and 242	~80	~6	175	No	~20
**Metasurface material**	Nb_2O_5 $+Sb_2S_3$	hBN $+TiO_2$	hBN	a-Si+Sb_2S_3	Au	Ag $+SiO_2$	Perovskite nanocrystals	GaN	Si QDs on metal
**Material crystallinity**	Amorphou s	Amorph ous	Crystall ine	Amorphous and Crystalline	Amorp hous	Amorph ous	Crystalline	Crysta lline	Crystalli ne

**Fluorescent medium**	CIS QDs hBN flake	hBN	hBN	nc-Si QDs	HgTe QDs	NV nano-diamond	Perovskite nanocrystals	GaN	CdSe/ZnS QDs
**PL amplification factor**	33	-	-	15	12	-	14	1	13
**PL tuning range (nm) via dimension sweep**	17	-	-	105	80	No	28	No	No
**PL tuning range (nm) via optical properties**	28	-	-	24	No	No	No	No	No
**SPE amplification factor**	222~2440	12.3	$>10^4$	No	No	No	No	No	No

References

(1) Do, T. T. H.; Nonahal, M.; Li, C.; Valuckas, V.; Tan, H. H.; Kuznetsov, A. I.; Nguyen, H. S.; Aharonovich, I.; Ha, S. T. Room-temperature strong coupling in a single-photon emitter-metasurface system. *Nature Communications* **2024**, *15* (1), 2281.
(2) Cao, S.; Jin, Y.; Dong, H.; Guo, T.; He, J.; He, S. Enhancing single photon emission through quasi-bound states in the continuum of monolithic hexagonal boron nitride metasurface. *Journal of Physics: Materials* **2021**, *4* (3), 035001.
(3) Abdelraouf, O. A.; Wu, M.; Liu, H. Hybrid Metasurfaces Enabling Focused Tunable Amplified Photoluminescence Through Dual Bound States in the Continuum. *Advanced Functional Materials* **2025**, 2505165.
(4) Sergeeva, K. A.; Pavlov, D. V.; Seredin, A. A.; Mitsai, E. V.; Sergeev, A. A.; Modin, E. B.; Sokolova, A. V.; Lau, T. C.; Baryshnikova, K. V.; Petrov, M. I. Laser-Printed Plasmonic Metasurface Supporting Bound States in the Continuum Enhances and Shapes Infrared Spontaneous Emission of Coupled HgTe Quantum Dots. *Advanced Functional Materials* **2023**, *33* (44), 2307660.
(5) Zheng, P.; Liang, L.; Arora, S.; Ray, K.; Semancik, S.; Barman, I. Pyramidal Hyperbolic Metasurfaces Enhance Spontaneous Emission of Nitrogen-Vacancy Centers in Nanodiamond. *Advanced optical materials* **2023**, *11* (6), 2202548.
(6) Aftenieva, O.; Brunner, J.; Adnan, M.; Sarkar, S.; Fery, A.; Vaynzof, Y.; König, T. A. Directional amplified photoluminescence through large-area perovskite-based metasurfaces. *ACS nano* **2023**, *17* (3), 2399-2410.
(7) Mohtashami, Y.; DeCrescent, R. A.; Heki, L. K.; Iyer, P. P.; Butakov, N. A.; Wong, M. S.; Alhassan, A.; Mitchell, W. J.; Nakamura, S.; DenBaars, S. P. Light-emitting metalenses and meta-axicons for focusing and beaming of spontaneous emission. *Nature communications* **2021**, *12* (1), 3591.
(8) Yang, G.; Niu, Y.; Wei, H.; Bai, B.; Sun, H.-B. Greatly amplified spontaneous emission of colloidal quantum dots mediated by a dielectric-plasmonic hybrid nanoantenna. *Nanophotonics* **2019**, *8* (12), 2313-2319.
